# Unraveling the genetic architecture of blood unfolded p-53 among non-demented elderlies: novel candidate genes for early Alzheimer's disease

**DOI:** 10.1186/s12864-024-10363-6

**Published:** 2024-05-03

**Authors:** Arash Yaghoobi, Seyed Amir Malekpour

**Affiliations:** School of Biological Sciences, Institute for Research in Fundamental Sciences (IPM), Tehran, 19395-5746 Iran

**Keywords:** Alzheimer’s disease, U-p53, GWAS, SNP, Single-cell RNA-seq, Single-cell ATAC-seq

## Abstract

**Background:**

Alzheimer's disease (AD) is a heritable neurodegenerative disease whose long asymptomatic phase makes the early diagnosis of it pivotal. Blood U-p53 has recently emerged as a superior predictive biomarker for AD in the early stages. We hypothesized that genetic variants associated with blood U-p53 could reveal novel loci and pathways involved in the early stages of AD.

**Results:**

We performed a blood U-p53 Genome-wide association study (GWAS) on 484 healthy and mild cognitively impaired subjects from the ADNI cohort using 612,843 Single nucleotide polymorphisms (SNPs). We performed a pathway analysis and prioritized candidate genes using an AD single-cell gene program. We fine-mapped the intergenic SNPs by leveraging a cell-type-specific enhancer-to-gene linking strategy using a brain single-cell multimodal dataset. We validated the candidate genes in an independent brain single-cell RNA-seq and the ADNI blood transcriptome datasets. The rs279686 between AASS and FEZF1 genes was the most significant SNP (*p*-value = 4.82 × 10^–7^). Suggestive pathways were related to the immune and nervous systems. Twenty-three candidate genes were prioritized at 27 suggestive loci. Fine-mapping of 5 intergenic loci yielded nine cell-specific candidate genes. Finally, 15 genes were validated in the independent single-cell RNA-seq dataset, and five were validated in the ADNI blood transcriptome dataset.

**Conclusions:**

We underlined the importance of performing a GWAS on an early-stage biomarker of AD and leveraging functional omics datasets for pinpointing causal genes in AD. Our study prioritized nine genes (SORCS1, KIF5C, TMEFF2, TMEM63C, HLA-E, ATAT1, TUBB, ARID1B, and RUNX1) strongly implicated in the early stages of AD.

**Supplementary Information:**

The online version contains supplementary material available at 10.1186/s12864-024-10363-6.

## Introduction

Dementia has a prevalence of about 7.1% among Europeans [1]. The most common type of dementia (50–70% of cases) is Alzheimer's disease (AD) [2]. AD is a progressive neurodegenerative disease whose asymptomatic phase begins decades before the onset of symptoms [3, 4]. AD is a multifactorial disease, and several risk factors, such as cardiovascular diseases, oral infection, sleep disturbance, aging, genetic susceptibility, traumatic brain injuries, and air pollution, have been proposed for it [5]. Regarding the beginning of the pathological process years before the onset of AD symptoms, early diagnosis of people at high risk for AD is pivotal.

Although the exact pathophysiological mechanisms of AD are not fully understood, the Amyloid-beta (Aβ) hypothesis has been the dominant one in describing AD pathogenesis. According to this hypothesis, aging causes excessive accumulation of Aβ peptide in the brain which eventually leads to the accumulation of toxic brain plaques and the onset of symptoms of AD. Genetic studies have also shown that in rare cases of AD, there are mutations in genes related to Aβ metabolism, strengthening the Aβ hypothesis [6]. However, this hypothesis has been subjected to a closer examination in recent years. Indeed, it has been questioned to some extent because new drugs based on antibodies against Aβ have poorly improved the symptoms of patients with mild and moderate forms of AD [7–9]. Therefore, there may be other biological pathways involved in the early stages of AD.

Based on the observed drawbacks of the Aβ hypothesis, various models have been proposed to describe the underlying mechanisms of AD. One of the proposed mechanisms for AD is a defect in the antioxidant response system and the role of the p53 protein in this process [10]. In addition to playing a role in tumor suppression in the body, the p53 protein can prevent the progress of neurodegeneration through various pathways, such as synaptic function, neurite outgrowth, protection from oxidative agents, and axonal regeneration [11–14]. For the first time in 2002, there were reports of observing a conformationally changed structure of p53 protein in the peripheral cells of patients with AD [15]. This protein, called unfolded p53 (U-p53), was not seen in other diseases, such as cancer and other neurodegenerative diseases, and seemed specific to AD. In the following years, it was suggested that the oxidative agents, chronic subtoxic oxidative stress, and sublethal Aβ concentrations may increase the expression of the U-p53 protein [16, 17]. It is worth noting that brain is an essential source of oxidative agents’ production since it consumes more than 20% of the body’s oxygen [18–20]. The rise of oxidative substances in the body causes various alterations in post-translational modification, the tertiary structure, and physiological functions of the p53 protein [15]. Moreover, the Aβ peptide can also cause similar changes in the p53 structure [21–23]. Conversely, the p53 protein can also affect Aβ concentration [24]. Additionally, oxidative agents can also lead to the phosphorylation of tau protein, which is one of the pathological hallmarks of AD. Hence, pathways related to oxidative stress could play key roles in the early stages of neurodegeneration [25, 26].

In recent years, there has been a growing interest in developing blood biomarkers for the early diagnosis of AD [27]. Developing biomarkers for AD has two main advantages: First, aiding in early diagnosis of the disease; Second, helping to develop new drugs for AD. The second advantage is precious since the development of effective drugs for AD has slowed, and the available drugs mostly have little effect on improving or delaying the symptoms of this disease [28]. However, most AD biomarkers have not yet entered the official diagnostic protocols of the disease [29]. Recently, an antibody has been developed to identify the blood U-p53 protein, which had a promising performance in identifying people at high risk for AD in the asymptomatic and symptomatic stages of the disease [30]. In a recent study, the blood U-p53 protein has been proposed as a robust biomarker outperforming other well-known AD biomarkers, such as Aβ PET scan, for identifying people at risk of developing AD 6 years before the onset of symptoms (AUC > 98%) [14].

According to several studies, the heritability of AD is estimated between 60–80% [31]. So far, multiple genome-wide association studies (GWAS) have been performed on AD, which have led to the discovery of numerous single nucleotide polymorphisms (SNPs) and genes [32]. However, the exact mechanism of the disease remains unknown. GWAS studies focusing on AD as a phenotype encounter numerous challenges due to two primary reasons: First, the clinical diagnostic accuracy of AD is poor. Second, due to the influence of age on AD, it is recommended to utilize age-matched control groups in GWASs conducted on this disease. However, the feasibility of obtaining such control groups is often limited [33]. To overcome these challenges, an alternative approach involves conducting GWAS on early-stage biomarkers of AD and utilizing these biomarkers as endophenotypes. This approach allows for the identification of novel genes and SNPs associated with the biological pathways underlying the early stages of the disease [34]. Compared to traditional GWASs performed on binary phenotypes, this approach has several advantages: First, continuous quantitative phenotypes, such as U-p53 concentration, represent real laboratory measurements rather than subjective binary clinical diagnoses. Second, this approach enhances the statistical power of GWAS even with smaller sample sizes compared to studies focusing on binary phenotypes. Third, early-stage endophenotypes could capture slight differences in susceptibility to disease that could not be detected with clinical binary phenotypes. So far, several GWASs have been conducted on various AD biomarkers, such as Aβ, tau protein, phosphorylated tau protein, and sTREM concentrations of cerebrospinal fluid (CSF), which has led to the discovery of novel genes and pathways involved in AD [34, 35].

Considering the role of the U-p53 protein in the early stages of AD and the high performance of this biomarker in predicting AD in the asymptomatic and early stages of this disease, we decided to conduct a GWAS on this biomarker as one of the AD endophenotypes to reveal novel genes and biological pathways involved in the early stages of AD. Our primary hypothesis was that the SNPs associated with blood U-p53 variation among older adults could shed light on novel loci involved in pathways related to the early stages of AD. By employing single-cell RNA-seq and multimodal datasets, as well as leveraging a single-cell enhancer-to-gene mapping strategy, we were able to establish connections between non-coding SNPs identified in our GWAS and their corresponding cell-specific target genes. This approach enabled us to gain deeper insights into the specific genes within brain cells that are causally implicated in the pathogenesis of AD.

## Methods

### Study design

We used the ADNI cohort for our research. In summary, ADNI is a cohort launched by Michael W. Weiner, MD, in 2003. The primary goal of this cohort is to gather various types of biomarkers, including body fluid biomarkers, neuroimaging, and genetic data, for developing tools for early diagnosis of AD. This cohort includes 3 phases: ADNI1, ADNIGO/2, and ADNI3. All samples have been collected using the relevant guidelines. A consent form has been obtained from all the cohort participants, and the regional ethics committees have also approved this cohort. The details of this cohort can be found on its website (http://adni.loni.usc.edu/). By personal communications, we had permission to access the database via the https://ida.loni.usc.edu/.

### Participants

To carry out our GWAS, we first used the file “DIADEM_V2_08_30_22.csv” which contains a dataset of U-p53 concentration in the blood plasma of 584 subjects from the ADNI GO/2 phase. The genotypic data of the ADNI GO/2 phase participants are available in 2 distinct PLINK files (“ADNI_GO_2_OmniExpress.zip” and “ADNI_GO2_2nd.tar.gz files”). The first file has the genotypic data of 432 subjects and the second one consists of the genotypic data of 361 subjects. In the next step, we merged these two files using the PLINK 1.9 software (https://www.coggenomics.org/plink/) which yielded a single file containing genotypic data of 793 subjects. We also used the “ADNIMERGE.csv” file which contains demographic data of the participants of the ADNI 1, GO/2, and 3 phases.

### Quality control and merging

To perform the GWAS, we conducted several stages of quality control on the samples with available genotypic information. We utilized ADNI GO/2 subjects genotyped by the Illumina HumanOmniExpress BeadChip in 2014. The genomic quality control steps were performed using the PLINK 1.9 software. First, we excluded participants with a missing genotype rate exceeding 5% and a heterozygosity rate deviating more than three standard deviations from the mean. Additionally, we assessed the concordance between participants' ascertained gender and the estimated inbreeding coefficient calculated by the PLINK software to identify potential gender mismatches. Next, we merged the output file with our phenotype file to extract subjects with both genotypic and phenotypic data. Subsequently, we excluded SNPs located on the sex chromosomes. SNPs with a minor allele frequency (MAF) less than 2%, a missing genotype rate exceeding 5%, and a Hardy–Weinberg equilibrium (HWE) *p*-value less than 10^–6^ were also excluded.

### Phenotype

Blood samples of 593 participants from the ADNI cohort were analyzed using the AlzoSure® Predict method to measure the blood plasma concentration of U-p53 protein, from June 16 to July 12, 2022, in more than eight rounds. In summary, the AlzoSure® Predict method is a diagnostic laboratory method that uses LC–MS/MS or Ion Trap on protein-depleted plasma samples. This method is particularly relevant for individuals over 50 years old with a family history of AD, a documented genetic predisposition, and signs of mild cognitive impairments (MCI). It aims to predict the progression from MCI to AD. During each round of analysis, approximately 54–77 samples from ADNI subjects were included. Additionally, at least three quality control samples from the Australian Imaging, Biomarkers and Lifestyle (AIBL) biobank, previously analyzed and covering the full range of U-p53 protein expression during the early stages of AD progression, were included as well. Out of the 593 samples, nine samples were discarded from the analysis despite undergoing two rounds of analysis due to the inability to obtain results. Detailed information regarding the methodology of laboratory tests and quality control procedures can be found on the ADNI website.

### Genome-wide association analysis

To account for population substructures as a potential confounding factor, we utilized the PLINK software to compute the principal components (PCs) of the genotypic data. The top 3 PCs were selected and incorporated as covariates in the analysis. This approach enabled us to control for genetic relatedness and address any potential biases introduced by population substructures [36]. To perform GWAS analysis, we used the linear regression model with the “–assoc –linear” commands in the PLINK 1.9 software. Also, we used age and gender as two other covariates. To calculate the significant and suggestive *p*-value thresholds, we used the “–indep –pairwise” command in the PLINK software (window size = 50 Kb, Linkage disequilibrium (LD) *r*^2^ > 0.8, and step size = 5) to calculate the number of independent SNPs. To evaluate the degree of genomic inflation for adjusting population substructures, we computed the λ-statistic. We also highlighted the observed versus expected *p*-values in the Q-Q plot using the qqman package in R. Power calculation was performed using the “genpwr” package in R. According to our calculation, our study has 80% power at the alpha = 2 × 10^–7^ (the significant threshold of our GWAS) to detect effect sizes of 0.14 and 0.32 when the MAF of SNPs is 0.50 and 0.05, respectively. Note that power is the probability of avoiding a type II error or false negative predictions, in which we are especially interested.

### Pathway analysis

We used the Pathway Scoring Algorithm (Pascal) software developed by David Lamparter et al. to perform pathway analysis using our GWAS summary statistic. In short, Pascal is fed with the SNP *p*-values derived from the GWAS analysis. We selected the “sum” option which takes the average of association signals (*p*-values) of all SNPs around ± 50 Kb of each gene. The maximum number of SNPs considered for averaging the chi-squared statistics of each gene was set to 3000. We set the MAF cut-off value to 0.01. We also used the European population of the 1000 Genome Project phase 3 for calculating LD. The merge distance option was set to 1. More details about the Pascal methodology have been described elsewhere [37]. We explored the KEGG, Biocarta, and Reactome pathway databases, to identify the biological pathways that are significantly enriched with the SNP-associated genes.

### Positional mapping of coding SNPs and step 1 fine mapping of intergenic SNPs using an AD single-cell gene program

Coding SNPs were simply annotated to their respective residing genes. For mapping intergenic SNPs, we employed a 2-step approach. At the step1, we first implemented a simple positional mapping approach by annotating intergenic SNPs to two nearest genes on the left and right sides of SNPs. Then, to produce a more AD-related comprehensive list of risk genes, we used an AD single-cell gene program constructed by Karthik A. Jagadeesh et.al. [38]. They used a single-nucleolus RNA-seq of the prefrontal cortex of 48 healthy and AD subjects from the ROS-MAP cohort omics project to construct this gene program. In summary, they computed a gene-level nonparametric Wilcoxon’s rank-sum statistic to identify the differentially expressed genes (DEGs), between cells from healthy and AD tissues for each cell type. Then, the gene weighting in the AD gene program was determined by transforming the gene *p*-values for each cell type. This transformation involved converting the *p*-values to X =  − 2log(*p*-value), which follows a χ2 distribution. The transformed values were then normalized to a grade between 0 and 1 using the min/max normalization formula g = (X – min(X))/(max(X) – min(X)). This process resulted in a relative weighting of genes in the AD gene program.

To associate our significant intergenic SNPs with nearby genes, we utilized the UCSC human genome build 38 and the MAGMA software (https://ctg.cncr.nl/software/magma). We annotated the SNPs to genes within a ± 500 Kb window. From the generated list of genes, we specifically selected those that overlapped with weights greater than 0.30 in any of the AD single-cell gene program for each cell type. These selected genes were then used for the subsequent fine mapping in step 2.

### Exploring SNPs with high LD at intergenic genomic risk loci

In order to identify all the SNPs in LD with our suggestive and significant intergenic SNPs, we used the FUMA software (https://fuma.ctglab.nl/) and NIH LDlink webtool (https://ldlink.nih.gov/?tab=ldproxy). In FUMA, the SNP2GENE function was utilized to identify SNPs in LD with our suggestive and significant intergenic SNPs of interest. Additionally, the LDlink webtool was utilized to assess LD using the European population of the 1000 Genome Project phase 3 as the reference genome panel. A threshold of *r*^2^ > 0.6 was chosen to determine high LD for SNPs, except for the major histocompatibility complex (MHC) region on chromosome 6, where a higher threshold of *r*^2^ > 0.9 was adopted due to its complex LD structure.

### Step 2 fine mapping of intergenic SNPs using an AD and healthy brain tissue multimodal dataset

In order to pinpoint potential risk genes more accurately, we sought to link our non-coding significant SNPs to their cell-type specific target genes using a brain tissue multimodal dataset. First, we downloaded a multimodal dataset isolated from post-mortem dorsolateral prefrontal cortex (DLPFC) tissues of seven AD and eight healthy controls from the GEO database (GSE214637). This dataset has simultaneously profiled the chromatin accessibility (single-cell assay for transposase-accessible chromatin with sequencing, or ATAC-seq) and gene expression (single-cell RNA-seq) from the same cells. More details about the samples of the original study can be found elsewhere [39]. We used the Seurat package to create an object containing both single-cell ATAC-seq and RNA-seq sparse matrices. In the next step, we used a recently introduced Single-cell Enhancer Target gene mapping (SCENT) method to link our significant intergenic SNPs to their target genes. In summary, the SCENT method applies the Poisson regression to model the effect of chromatin accessibility on gene expression in order to identify potential enhancers and silencers for each gene in a cell-type specific manner. More details about the SCENT package can be found elsewhere [40]. Our primary hypothesis was that our significant non-coding SNPs or their proxy SNPs might be located in enhancers or silencers and regulate the expression of potential risk genes. So, we first explored the intersection of position of our significant SNPs or their proxies with ATAC-seq peaks using the UCSC human genome build 38 version. Then at each intersected region, we used the SCENT package to investigate the potential association of that ATAC-seq peak region signals with the expression level of candidate genes produced in the step 1 fine mapping. We also used log-normalized RNA counts, percentage of mitochondrial reads in each cell, age, and gender as covariates. The *p*-value less than 0.05 was chosen as the threshold indicating significant results.

### Validating differential expression of final mapped genes between AD and healthy controls using an independent single-cell RNA-seq dataset

We obtained a single-cell RNA-seq dataset from an independent cohort with accession number GSE174367. The dataset consists of nuclei isolated from the prefrontal cortex of post-mortem brain tissues, including 11 late-stage AD samples and seven age-matched healthy control samples. The original study by Samuel Morabito et al. [41] provides complete details on this dataset. Briefly, the MAST package (v1.12.0) was applied to identify DEGs between AD and control samples in this single-cell RNA-seq dataset. The resulting DEG list was generated, with *p*-values adjusted using the Bonferroni correction method. Supplementary data 1 of Samuel Morabito et al. [41] also provides the DEG list between AD and control samples. We proceeded to identify the genes that were common between the DEGs from Samuel Morabito et al. [41] and our final mapped genes obtained from the previous steps.

### Validating association of the mapped genes with blood U-p53 at transcription level using ADNI whole blood transcriptomics

To validate some of the potential genes associated with the concentration of U-p53 and possibly early AD, we performed a linear regression analysis to examine the relationship between the concentration of U-p53 (dependent variable) and the expression of our candidate genes (independent variables), in blood. Age and gender were included as covariates in the analysis. The regression model can be represented as follows:

1 $$\mathrm U-\mathrm p53\;\mathrm{concentration}\;\sim\;\mathrm\alpha\;+\;\mathrm\beta1\;\times\;\mathrm{candidate}\;\mathrm{gene}\;\mathrm{expression}\;+\;\mathrm\beta2\;\times\;\mathrm{age}\;+\;\mathrm\beta3\;\times\;\mathrm{sex}$$

For each candidate gene, Eq. (1) was fitted to the U-p53 concentration and candidate gene expression data obtained from the ADNI dataset. To achieve this, we downloaded the “Microarray_Gene_Expression_Profile_Data.csv” file from the ADNI cohort which contains microarray transcriptome of 811 participants from the ADNI 1 and GO/2 phases. Briefly, the transcriptome profiling of 811 participants was performed at Bristol-Myers Squibb (BMS) laboratory using the peripheral blood samples. The Affymetrix Human Genome U219 Array (www.affymetrix.com) was used to determine the transcriptome profile. More details about the quality control steps and laboratory methods can be found on the ADNI website. When fitting Eq. (1) to the gene expressions from ADNI, the Wald statistic was calculated for the β1 as the coefficient of the candidate gene expression. The *p*-value, derived from the Wald statistic, was then used to assess the significance of the association, with a threshold of *p*-value less than 0.01 considered as indicative of significance.

**Fig. 1:**
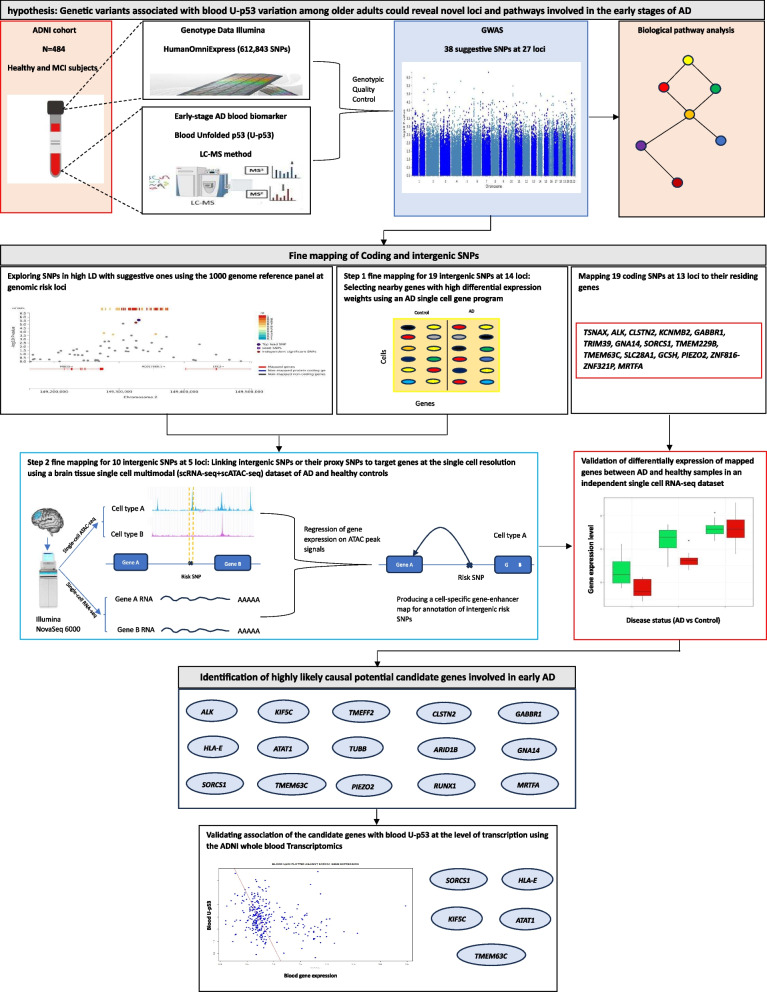
Graphical depiction of all of the steps performed in the study. The figure shows the GWAS pipeline used in our study. The upper part of the figure shows the datasets, GWAS and pathway analyses. The middle part of the pipeline shows the steps for positional and functional fine-mapping of SNPs using brain single-cell RNA-seq and multimodal datasets. Lastly, the lower part of the figure shows the validated candidate genes in the independent single-cell RNA-seq and ADNI blood microarray transcriptome datasets

The all steps performed in our study are graphically depicted in Fig. 1.

## Results

### Quality control and genome wide association results

**Fig. 2:**
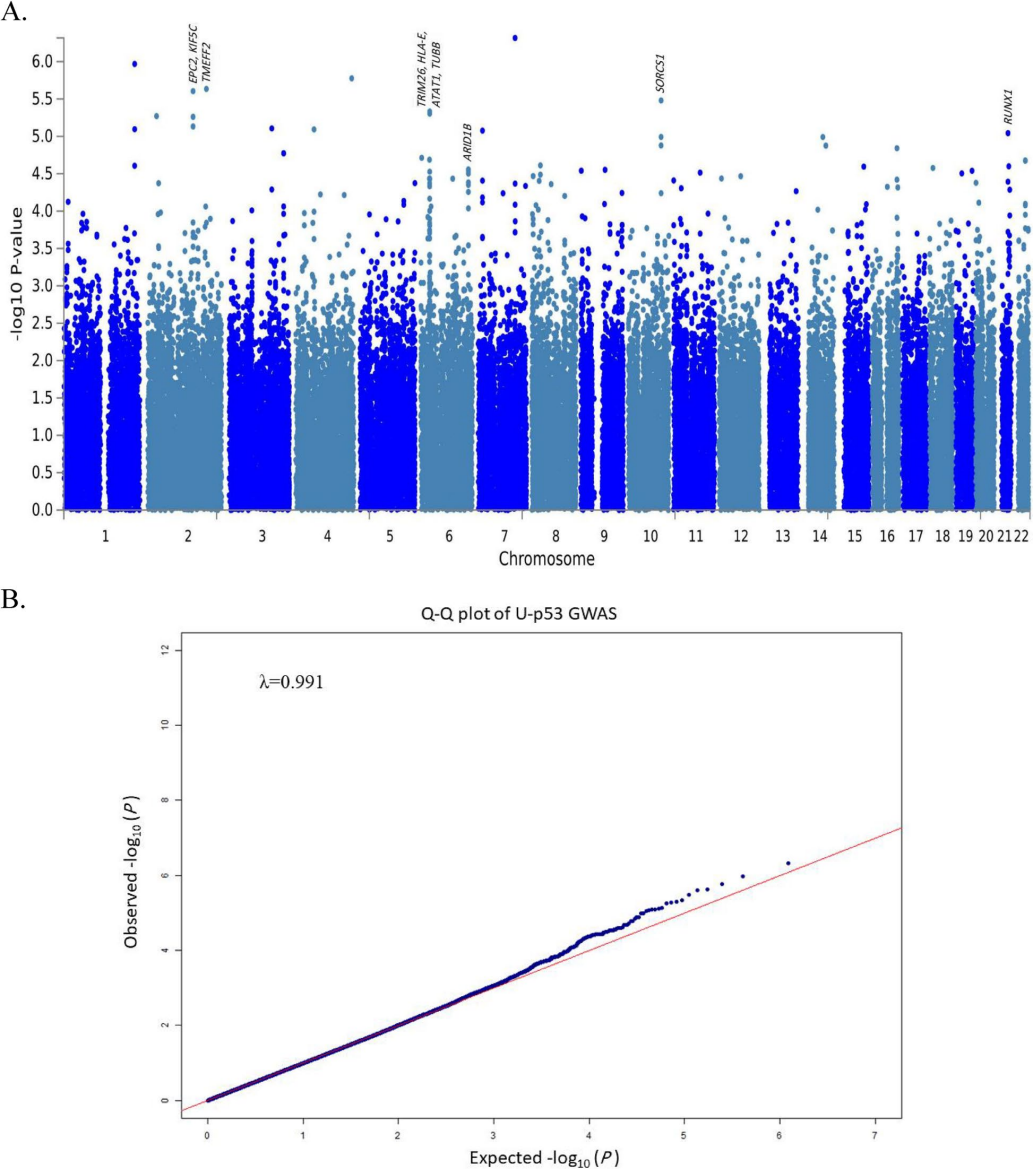
Manhattan and Q-Q plot of the blood U-p53 GWAS. **A** Manhattan plot of the blood U-p53 GWAS. Only six suggestive loci with strong evidence of involvement in AD pathogenesis in our analyses are shown. **B** Q-Q plot of the blood U-p53 GWAS results showing low genomic inflation due to the population substructures (λ = 0.99184)

Merging two genotypic files of the ADNIGO/2 phase yielded genotypic data of 793 subjects (425 men and 368 women) on 730,525 SNPs. Twenty participants were excluded due to a heterozygosity rate of more than three standard deviations from the mean. There was no discordance between the subject’s ascertained gender information with gender estimation based on genotypes. Missing genotype rates of all participants were less than 5%. Among 773 remaining subjects, 484 were kept since they had both genotypic and blood U-p53 data. 79,777 SNPs with MAF of less than 2% were excluded. 16,483 SNPs were excluded due to a missing genotype rate of more than 5%. 255 SNPs with Hardy–Weinberg *p*-value less than 10^–6^ were also excluded. 21,167 SNPs on sex chromosomes were also excluded. Finally, the genotypic data of 612,843 SNPs among 484 participants passed all the quality control steps. The total genotypic rate was 0.9986. Among the 484 participants of our study, there were 190 cognitively normal (CN) subjects (121 healthy subjects and 69 subjects with subjective memory complaints) and 363 patients with MCI (200 subjects with early mild cognitive impairments (EMCI) and 94 subjects with late mild cognitive impairments (LMCI)). The mean age of our study participants was 71.78 (± 6.97). Finally, GWAS was performed on 484 subjects (260 male and 224 female). The λ-statistic indicating the degree of genomic inflation due to population structures was low in our analysis (λ = 0.99184). The Manhattan and Q-Q plot of blood U-p53 GWAS are shown in Fig. 2. To check the robustness of our GWAS results, we performed linear mixed modeling to account for genetic relatedness using the Genome-wide Complex Trait Analysis (GCTA) software [42] (https://yanglab.westlake.edu.cn/software/gcta/#Overview), following the approach suggested by Junhao Wen et al. in their study [43]. The results obtained from the GCTA software align accurately with our final reported results obtained from the PLINK software. The GWAS summary statistics obtained from both software are provided in the Supplementary File 1.

We retained the independent SNPs by excluding 188,013 dependent SNPs (LD *R*^2^ > 0.8 at 50 Kb window) from the total SNPs. Then, our GWAS's significant and suggestive thresholds were calculated based on the number of independent SNPs (424,830 SNPs). So, we chose 2 × 10^–7^ (~ 0.05/424,830) and 3 × 10^–5^ (~ 200 × 0.05/424,830) as significant and suggestive *p*-value thresholds for our GWAS.

Among 612,843 SNPs, 38 were considered suggestive SNPs (2 × 10^–7^ < *p*-value < 3 × 10^–5^). These 38 suggestive SNPs were located at 27 genomic risk loci. None of the SNPs reached the significant threshold level (*p*-value < 2 × 10^–7^). The rs279686 had the smallest *p*-value and nearly reached the significant threshold (*p*-value = 4.82 × 10^–7^). Among 38 suggestive SNPs, 19 were intergenic.

### Pathway analysis results

**Fig. 3:**
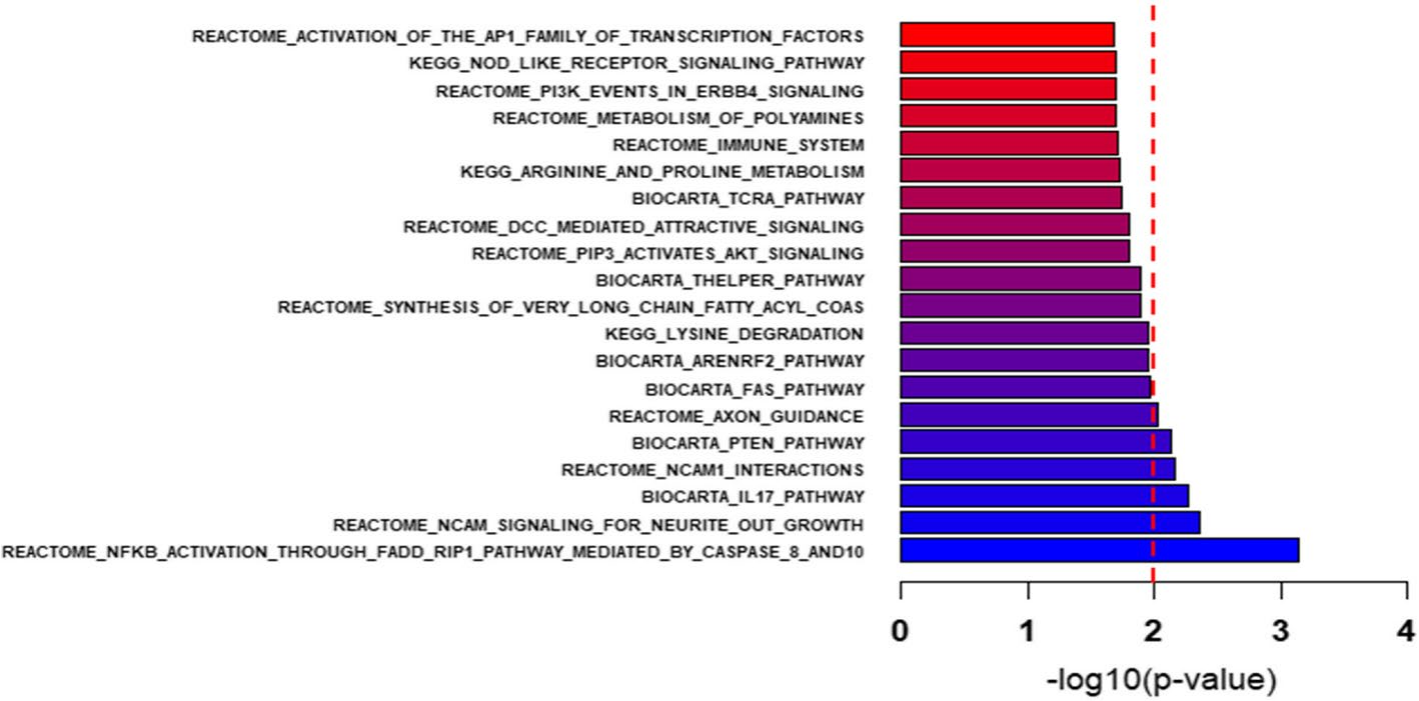
Bar plot for the − log10 of the *p*-value of selected pathways that are enriched with SNP-associated genes. A − log10(*p*-value) greater than 2 corresponds to pathways that are significantly enriched, with corresponding *p*-values less than 0.01, as determined using the Pascal package

The GWAS results provided genome-wide SNP *p*-values, which were subsequently used in Pascal package for gene scoring and pathway enrichment analysis. Pascal aggregates SNP signals within ± 50 Kb of each gene and employs these per-gene aggregated signals for the pathway enrichment analysis. In brief, we examined the KEGG, Biocarta, and Reactome pathways to identify significant enrichments of genes associated with SNPs. For more information see Methods. Our pathway analysis revealed six suggestive biological pathways associated with U-p53, with *p*-values below 0.01, as plotted in Fig. 3. Top biological pathway was REACTOME_NFKB_ACTIVATION_THROUGH_FADD_RIP1_PATHWAY_MEDIATED_BY_CASPASE_8_AND10 (*p*-value = 7.16 × 10^–4^). The other 5 suggestive pathways were REACTOME_NCAM_SIGNALING_FOR_NEURITE_OUT_GROWTH (*p*-value = 4.32 × 10^–3^), BIOCARTA_IL17_PATHWAY (*p*-value = 5.47 × 10^–3^), REACTOME_NCAM1_INTERACTIONS (*p*-value = 6.85 × 10^–3^), BIOCARTA_PTEN_PATHWAY (*p*-value = 7.27 × 10^–3^), and REACTOME_AXON_GUIDANCE (*p*-value = 9.46 × 10^–3^).

### Positional mapping of coding SNPs and step 1 fine mapping results

First positional mapping of coding SNPs was performed based on the human genome hg38 version, which resulted in mapping 15 genes. In the next step, positional mapping and functional gene mapping of intergenic SNPs using the AD single-cell gene program yielded 36 genes in various brain cell types. The results of all suggestive SNPs and their step 1 mapped genes are summarized in Table 1.

**Table 1:** Blood U-p53 GWAS summary statistics of suggestive SNPs and their potential candidate genes

Locus number	SNP	Ref. allele	Alt. allele	Chr	Frequency	*p*-value	Position	Candidate genes based on position (Intragenic SNPs)	Candidate genes based on AD single-cell gene program (intergenic SNPs)
1	rs6673974	A	G	1	0.04	2.47 × 10^–5^	EGLN1-TSNAX	-	EGLN1
1	rs7519365	A	G	1	0.04	1.075 × 10^–6^	TSNAX	TSNAX	-
1	rs11122309	T	C	1	0.04	8.031 × 10^–6^	TSNAX-DISC1	-	EGLN1
2	rs10171094	C	T	2	0.03	5.365 × 10^–6^	ALK	ALK	-
3	rs3936439	A	G	2	0.33	7.373 × 10^–6^	MBD5-EPC2	-	EPC2, KIF5C, MBD5
3	rs16828910	T	G	2	0.32	5.48 × 10^–6^	MBD5-EPC2	-	EPC2, KIF5C, MBD5
3	rs921243	T	G	2	0.33	2.481 × 10^–6^	MBD5-EPC2	-	EPC2, KIF5C, MBD5
4	rs4853647	G	A	2	0.09	2.317 × 10^–6^	CAVIN2-TMEFF2	-	TMEFF2
5	rs4462908	C	T	3	0.05	7.825 × 10^–6^	CLSTN2	CLSTN2	-
6	rs4624543	G	A	3	0.42	1.69 × 10^–5^	KCNMB2	KCNMB2	-
6	rs9844737	G	A	3	0.42	1.69 × 10^–5^	KCNMB2	KCNMB2	-
7	rs1482699	T	C	4	0.06	8.079 × 10^–6^	IGFBP7-ADGRL3	-	ADGRL3
8	rs10520544	C	A	4	0.04	1.674 × 10^–6^	DCTD-WWC2	-	TENM3
9	rs9405684	T	C	6	0.13	1.94 × 10^–5^	FAM50B-PRPF4B	-	-
10	rs3025642	C	T	6	0.05	2.05 × 10^–5^	GABBR1	GABBR1	-
11	rs9393999	A	G	6	0.14	2.97 × 10^–5^	TRIM26-TRIM39	-	-
11	rs12174823	T	C	6	0.12	4.958 × 10^–6^	TRIM26-TRIM39	-	-
11	rs2240058	A	G	6	0.12	4.653 × 10^–6^	TRIM39	TRIM39	-
12	rs2444783	A	G	6	0.14	2.89 × 10^–5^	NOX3-ARID1B	-	ARID1B
12	rs2250759	C	T	6	0.13	2.77 × 10^–5^	NOX3-ARID1B	-	ARID1B
13	rs2159808	C	A	7	0.30	8.384 × 10^–6^	DGKB-AGMO	-	DGKB
14	rs279686	A	G	7	0.27	4.82 × 10^–7^	AASS-FEZF1	-	PTPRZ1
15	rs4732990	C	T	8	0.15	2.44 × 10^–5^	DUSP4-SARAF	-	SARAF
16	rs1158989	A	G	9	0.27	2.88 × 10^–5^	SMARCA2-VLDLR	-	SMARCA2, VLDLR, KCNV2
17	rs7860549	A	G	9	0.07	2.80 × 10^–5^	GNA14	GNA14	-
18	rs7071222	A	G	10	0.11	1.32 × 10^–5^	SORCS1	SORCS1	-
18	rs2245123	T	C	10	0.09	1.02 × 10^–5^	SORCS1	SORCS1	-
18	rs17121635	A	G	10	0.10	3.307 × 10^–6^	SORCS1	SORCS1	-
19	rs17184650	C	T	14	0.11	1.02 × 10^–5^	TMEM229B	TMEM229B	-
20	rs1508299	T	G	14	0.02	1.33 × 10^–5^	TMEM63C	TMEM63C	-
21	rs7166440	T	C	15	0.04	2.54 × 10^–5^	SLC28A1	SLC28A1	-
22	rs12444974	G	A	16	0.07	1.44 × 10–5	GCSH	GCSH	-
23	rs196956	A	G	18	0.25	2.65 × 10–5	PIEZO2	PIEZO2	-
24	rs8109900	C	T	19	0.21	2.89 × 10–5	ZNF816-ZNF321P	ZNF816-ZNF321P	-
25	rs2835119	A	G	21	0.02	9.048 × 10–6	RUNX1-SETD4	-	RUNX1, SETD4, CBR1
26	rs3787909	G	A	21	0.03	2.51 × 10^–5^	ERG-ETS2	-	ETS2
27	rs6001930	T	C	22	0.09	2.11 × 10^–5^	MRTFA	MRTFA	-
27	rs6001931	G	A	22	0.09	2.11 × 10^–5^	MRTFA	MRTFA	-

### Step 2 fine mapping results

None of our suggestive intergenic SNPs were located in ATAC peaks, but using LD information at the suggestive intergenic loci, we could identify seven loci out of 14 suggestive loci that contain at least one SNP that is both in LD with the suggestive SNPs (*r*^2^ > 0.6) and is located in ATAC peaks of the multimodal dataset used in our analysis. At each seven loci, we investigated the association of each identified ATAC peak with the expression level of the candidate genes obtained from step 1 fine mapping, at each eight brain cell types using the SCENT method. At five loci out of the seven, we could map the suggestive non-coding SNPs to their putative target risk genes in five brain cell types. The fine-mapped genes and their target cell-type resulted from this step were: *EPC2* (*p*-value = 0.01 in Microglial cells and 3.62 × 10^–6^ in Oligodendrocyte progenitor cells (OPCs)), *KIF5C* (*p*-value = 8.94 × 10^–3^ in OPCs), *TMEFF2* (*p*-value = 1.33 × 10^–11^ in excitatory neurons), *ARID1B* (*p*-value = 0.002 in excitatory neurons), *HLA-E* (*p*-value = 0.03 in Microglial cells), *ATAT1* (*p*-value = 0.01 in excitatory neurons), *TRIM26* (*p*-value = 0.02 in excitatory neurons), *TUBB* (*p*-value = 0.001 in OPCs, 0.01 in Astrocytes, and 0.02 in inhibitory neurons), and *RUNX1* (*p*-value = 0.002 and 0.03 in Microglial cells). At loci 7 and 16, expression of no candidate genes was significantly associated with the ATAC peak signal of that region. The results of the significant genes with their related cell types are summarized in Table 2.

**Table 2:** SCENT package Summary statistics of blood U-p53 candidate genes at intergenic risk loci

Locus num	Significant Genes	Suggestive SNPs	Proxy SNPs	LD R^2^	ATAC peak region	SCENT *p*-value	Brain cell types
3	EPC2	rs3936439	rs11687034	0.77	Chr2:148,614,153–148,614,910	3.62 × 10^–6^	OPC
3	EPC2	rs3936439	rs11687034	0.77	Chr2:148,614,153–148,614,910	0.01	Microglia
3	KIF5C	rs3936439	rs11687034	0.77	Chr2:148,614,153–148,614,910	8.94 × 10^–3^	OPC
4	TMEFF2	rs4853647	rs72912394, rs72912395	0.68	Chr2:191,864,620–191865537	1.33 × 10^–11^	Excitatory
11	HLA-E	rs12174823	rs9391807, rs9391806	0.91	Chr6:30,259,185–30260006	0.03	Microglia
11	ATAT1	rs12174823	rs9391807, rs9391806	0.91	Chr6:30,259,185–30260006	0.01	Excitatory
11	TUBB	rs12174823	rs9391807, rs9391806	0.91	Chr6:30,259,185–30260006	0.02	Inhibitory
11	TUBB	rs12174823	rs9391807, rs9391806	0.91	Chr6:30,259,185–30260006	0.01	Astrocyte
11	TUBB	rs12174823	rs9391807, rs9391806	0.91	Chr6:30,259,185–30260006	0.001	OPC
11	TRIM26	rs12174823	rs9391807, rs9391806	0.91	Chr6:30,259,185–30260006	0.02	Excitatory
12	ARID1B	rs2250759	rs2603438	0.87	Chr6:157,507,177–156507907	0.002	Excitatory
25	RUNX1	rs2835119	rs73365738	0.96	Chr21:35,720,219–35721068	0.002	Microglia
25	RUNX1	rs2835119	rs12481979	0.81	Chr21:35,696,977–35,697,804	0.03	Microglia

### Validating differential expression of final mapped genes between AD and healthy controls in an independent single-cell RNA-seq dataset results

There were 15 genes common between our final 23 mapped genes and the DEG list obtained from the Morabito et al. [41] dataset. These 15 genes included: *ALK*, *KIF5C*, *TMEFF2*, *CLSTN2*, *GABBR1*, *HLA-E*, *ATAT1*, *TUBB*, *ARID1B*, *GNA14*, *SORCS1*, *TMEM63C*, *PIEZO2*, *RUNX1*, and *MRTFA*.

### Validating association of the mapped genes with blood U-p53 at the transcription level in ADNI whole blood transcriptome dataset results

By merging ADNI whole blood microarray transcriptome dataset with the U-p53 data, 322 subjects had both blood transcriptome and the blood U-p53 data. All probes of the 15 genes associated with blood U-p53 obtained from previous mapping steps were derived from ADNI whole blood transcriptome microarray dataset to investigate their possible association of expression level with blood U-p53. Among these genes, five genes were replicated at the blood transcript level. These 5 genes were *SORCS1* (*p*-value = 2.89 × 10^–5^), *HLA-E* (*p*-value = 4.37 × 10^–4^), *ATAT1* (*p*-value = 2.20 × 10^–3^), *KIF5C* (*p*-value = 2.38 × 10^–3^), and *TMEM63C* (*p*-value = 9.93 × 10^–3^).

## Discussion

**Fig. 4:**
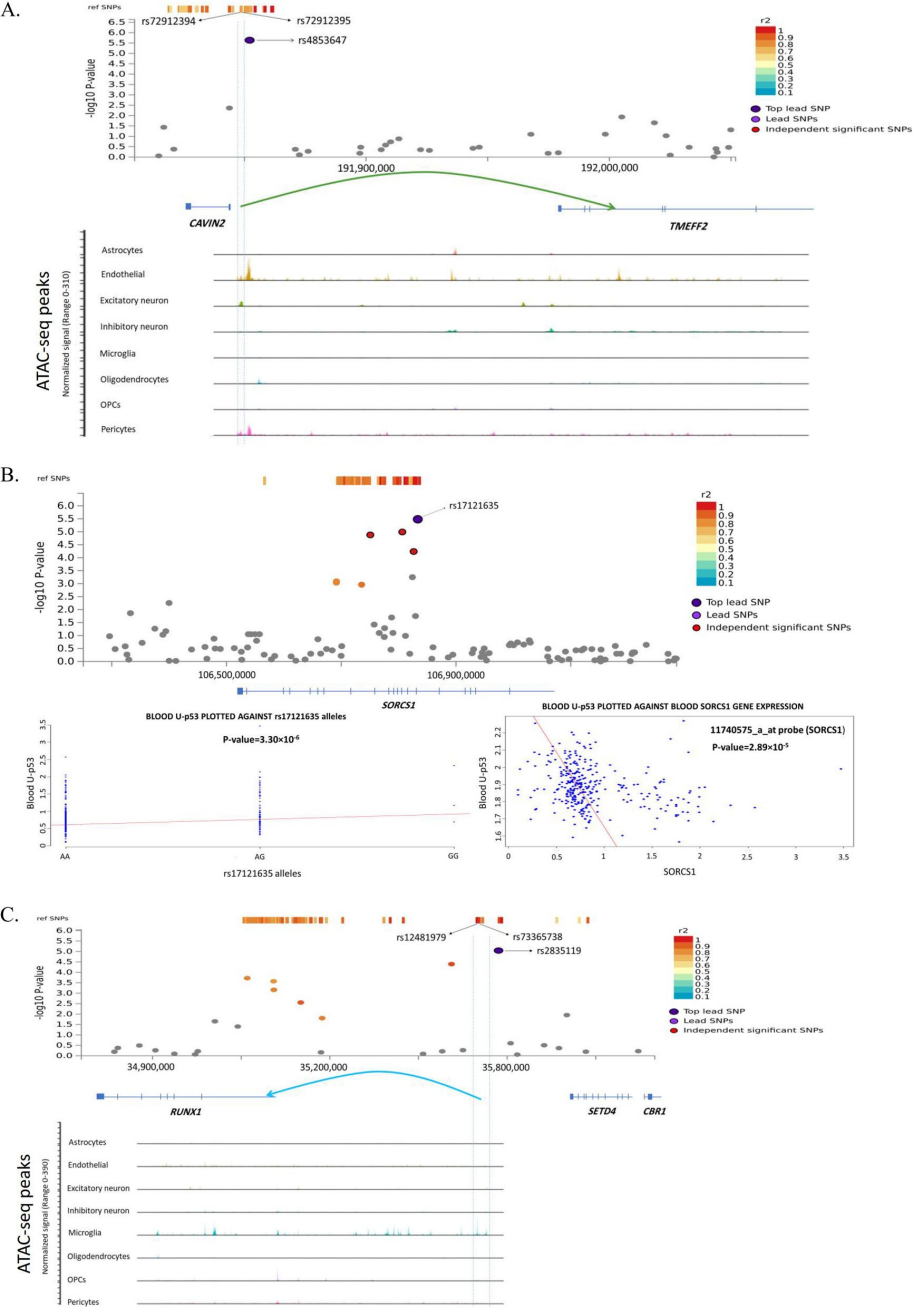
Graphical illustration of GWAS results at multiple levels across three loci with strong evidence of their involvement in AD pathogenesis. **A** The locus number 4 between *CAVIN2* and *TMEFF2* genes showing two SNPs (rs72912394, rs72912395) with LD *r*^2^ = 0.68 with the suggestive SNP (rs4853647) located within ATAC peak regions across endothelial, excitatory neuron, and pericyte cells. Our analysis revealed that this regulatory intergenic region is a potential silencer of the *TMEFF2* gene in excitatory neuron cells. **B** The locus number 18 shows the suggestive SNP (rs17121635) within the *SORCS1* gene. The left bottom picture shows the risk effect of the alternative allele G of this polymorphism by increasing the U-p53 concentration in blood. The right bottom picture shows the significant inverse association between the blood *SORCS1* gene expression and the blood U-p53. **C** The locus number 25 showing two SNPs (rs73365738, rs12481979) with LD *r*^2^ = 0.96 and LD *r*^2^ = 0.81 with the suggestive SNP (rs2835119) located within two distinct ATAC peak regions in the microglia cells. Our analysis revealed that both intergenic regions regulate the expression of the *RUNX1* gene in microglia cells

To our knowledge, this is the first GWAS on blood U-p53 as one of AD's novel, specific and sensitive biomarkers. Using a single-cell multimodal and two single-cell RNA-seq datasets for fine mapping of risk SNPs, we prioritized *KIF5C*, *EPC2*, *TMEFF2*, *HLA-E*, *ATAT1*, *TUBB*, *ARID1B*, and *RUNX1* as potential risk genes with strong evidence of involvement in the early stages of AD. We also suggested the *SORCS1* gene as the other important gene involved in AD, with robust evidence at genotypic and blood transcript levels. Aβ processing (*SORCS1* and *TMEFF2*) and axonal transportation (*KIF5C*, *TUBB*, *ATAT1*) were two potential common biological pathways among the identified genes. A graphical illustration of results of multiple analyses sources across three loci are shown in Fig. 4.

We identified six suggestive biological pathways associated with blood U-p53 and possibly involved in AD. Two of them (NFKB activation through FADD and RIP1 pathway mediated by CASPASE 8 and 10 and IL17 pathway) highlight the importance of immune system involvement in AD. Indeed, the NFKB is an inflammatory transcription factor that has important roles in AD pathogenesis [44]. Interestingly, another two suggestive pathways were related to the nervous system (NCAM signaling for neurite outgrowth and Axon guidance). We identified three SNPs at locus 18, residing in the *SORCS1* gene. Additionally, there was a strong and inverse association between the *SORCS1* gene blood expression and blood U-p53 (*p*-value = 2.89 × 10^–5^). *SORCS1* belongs to the Sortilin family of vacuolar protein sorting-10 (Vps10) domain-containing proteins. The *SORL1* gene, one of the other genes of this family, is one of the most famous and replicated loci in multiple AD GWASs to date [45]. Wei Xu et al. suggested that rs10884402 and rs950809 in intron 1 of the *SORCS1* gene were associated with late-onset AD in the Chinese Han population [46]. It has been shown that the *SORCS1* gene alters the Amyloid beta precursor protein (APP) processing [47]. It also has been shown that overexpression of the *SORCS1* gene in cultured cells lowers Aβ generation [48]. Recently, Alfred Kihoon Lee et al. proposed an important role for *SORCS1* in rescuing Aβ oligomers-induced Neurexin dysfunction and synaptic pathology [49].

The rs4853647 SNP at locus four was significantly associated with blood U-p53 in our study. This SNP resides between *CAVIN2* and *TMEFF2* genes. By using enhancer-to-gene linking strategy, it was revealed that this region was strongly associated with the *TMEFF2* gene expression in excitatory neurons (*p*-value = 1.33 × 10^–11^). *TMEFF2* (Transmembrane Protein With EGF Like And Two Follistatin Like Domains 2) gene belongs to the tomoregulin family of transmembrane proteins. Based on the Protein atlas database, it is highly expressed in brain and prostate tissues. Although, to date, this locus has never been reported to be associated with AD in previous GWASs, Hyun-Seok Hong et al., for the first time, showed that the TMEFF2 protein is related to Aβ metabolism and toxicity and could protect neurons from Aβ-induced toxicity [50]. This example highlights the power of an endophenotype-based GWAS in identifying novel loci previously reported to be related to the disease mechanisms in experimental studies.

At locus 3, there were three suggestive intergenic SNPs between the *MBD5* and *EPC2* genes. Interestingly, in a 2011 GWAS on AD CSF biomarkers among ADNI cohort subjects, the rs4499362 SNP between *EPC2* and *KIF5C* genes at this locus was associated with CSF total tau protein [51]. The authors proposed the EPC2 gene as a novel gene involved in AD pathogenesis. This locus contains three genes (*MBD5*, *EPC2*, *KIF5C*), all highly expressed in the brain, making fine-mapping of this locus more challenging. Using the enhancer-to-gene linking approach, we could map the suggestive region to *KIF5C* and EPC2 genes in OPC and *EPC2* gene in microglial cells. However, unlike the *KIF5C* gene, the *EPC2* gene was not differentially expressed between AD and healthy controls in an independent RNA-seq dataset. Additionally, our analysis revealed that there was a significant direct association between blood expression of the *KIF5C* gene and blood U-p53. So, these two genes may be involved in AD pathogenesis in a cell-specific manner. Although the exact function of the *EPC2* (enhancer of polycomb homolog 2) gene has not been fully characterized, it may relate to AD pathogenesis through the regulation of epigenetic mechanisms and chromatin remodeling [51]. The *KIF5C* (Kinesin Family Member 5C) is required for anterograde axonal transportation. Axonal transportation defects play important roles in AD pathophysiology [52].

We identified three SNPs along with many SNPs which had subthreshold *p*-values at locus 11. We could fine-map this region to 4 genes across various brain cell types; 3 (*HLA-E*, *ATAT1*, *TUBB*) were differentially expressed between AD and healthy cells. Interestingly, *ATAT1* and *TUBB* are involved in axonal transportation. *HLA-E* is involved in immune pathways. Interestingly, the blood expression of both *ATAT1* and *HLA-E* genes were also significantly associated with blood U-p53. This example highlights the challenges of fine mapping of GWAS SNPs and complexity of potential shared enhancers regulating multiple genes in different cell types in some of the risk loci identified in GWASs. Future studies based on CRISPR technology may unravel this complexity and pinpoint the true causal genes.

At locus 12, we identified two SNPs between *NOX3* and *ARID1B* genes. In a study in 2014, V K Ramanan et al. showed that the rs938448 SNP at this locus was associated with amyloid deposition in the brain [53]. Although at first glance, the *NOX3* gene seems to be the potential causal gene due to its involvement in oxidative agents’ production, our single-cell enhancer-to-gene linking analysis revealed that the region is significantly associated with the expression of the *ARID1B* gene in excitatory neurons. *ARID1B* (AT-Rich Interaction Domain 1B) gene is a subunit of the mammalian SWI/SNF complex. It has an important role in chromatin remodeling. Although the exact mechanisms of this gene in AD pathogenesis are unclear, *ARID1B* mutations have been reported as monogenic causes of autism spectrum disorder (ASD) and intellectual disabilities. It has been shown that *ARID1B* haploinsufficiency can impair inhibitory synaptic function and increase excitation to inhibition (E/I) balance in the brain [54]. This example highlights the challenges of finding target genes of intergenic SNPs based solely on potential disease-related biological mechanisms.

At locus 25, the rs2835119 SNP, located between *RUNX1* and *SETD4* gene, was associated with blood U-p53. Analyzing two different open chromatin regions harboring two SNPs with high LD with the rs2835119 revealed that both regions were potential enhancers and silencers of the *RUNX1* gene in microglial cells. Ashok Patel et al. showed that variants (rs4816501) in *RUNX1* were associated with AD among patients with Down syndrome [55]. The *RUNX1* (RUNX Family Transcription Factor 1) gene encodes a transcription factor regulating the expression of numerous genes involved in normal hematopoiesis. It has been shown that *RUNX1* is involved in the regulation of TLR1/2 and TLR4 signaling pathways and inflammatory cytokine production [56]. The relevance of this gene in AD pathogenesis may be through immune system pathways. Future experimental studies are needed to uncover this.

Among intragenic SNPs that their relevant genes were differentially expressed between AD and healthy cells, products of some genes like *GABBR1* and *CLSTN2* are located in synapses. Among the other genes, it has been shown that overexpression of the *MRTFA* gene reduces the accumulation of Aβ peptide [57]. Heterozygous deletion of the other gene, *GNA14*, has been reported in an early-onset AD patient [58]. By studying the 3xTg-AD and tauC3 mouse AD models, Jisu Park et al. proposed that the *ALK* gene has a crucial role in tau-mediated neurodegeneration [59].

At the proteomics level, we investigated whether there are significant differences in the protein levels of our proposed genes between the brain tissues of AD patients and control subjects. To achieve this, we utilized the NeuroPro database (https://neuropro.biomedical.hosting/) [60], which has compiled a comprehensive list of differentially expressed proteins by conducting a meta-analysis of 38 brain proteomic studies across different stages of AD. Among the 15 proposed genes in our final list, protein levels of six genes were differentially expressed between brain tissues of AD and control subjects. Interestingly, the protein levels of TMEFF2 and TMEM63C were upregulated in the frontal cortex of patients with preclinical stages of AD compared to the controls [61]. This suggests that TMEFF2 and TMEM63C may serve as novel target genes for early-stage intervention and should be further investigated in future studies, as they have received less attention in the context of AD. The other four proteins, including the SORCS1, CLSTN2, TUBB, and GABBR1, were differentially expressed between patients with AD and healthy controls.

Although using a quantitative phenotype in our GWAS increased the power of our study, due to their high cost and laboratory challenges, GWASs on endophenotypes always suffer from relatively small sample sizes compared to traditional GWASs [45]. Future studies are needed to increase the sample sizes for various AD biomarkers, such as U-p53. However, our study highlights the power of leveraging two approaches in AD GWASs: First, it showed using a powerful early-stage biomarker of AD as an endophenotype in AD GWASs can lead to identifying novel loci and genes involved in the early stages of AD. Second, it showed that integration of GWAS results with single-cell datasets and leveraging powerful enhancer-to-gene linking strategies can pinpoint the true causal genes in a cell-specific manner.

## Conclusions

In conclusion, through the integration of GWAS results on U-p53 as a reliable early-stage AD biomarker with brain single-cell RNA-seq and multimodal datasets, we have identified several significant genes associated with blood U-p53 and potentially with AD, particularly among non-demented subjects. These genes include SORCS1, TMEFF2, TMEM63C, KIF5C, HLA-E, TUBB1, ATAT1, ARID1B, and RUNX1. We believe that these proposed candidate genes hold substantial value for further exploration in future experimental studies, both in terms of their functional and structural aspects, due to their potential roles in the early stages of AD. For instance, genes like TMEFF2 and TMEM63C have received comparatively less attention in the context of AD, but there are some evidences indicating that their protein expression levels are altered in the preclinical stages of the disease. This highlights the importance of investigating these genes further. Furthermore, our results could provide valuable insights for the design of future drug targets.

Given the limited availability of cohorts where blood U-p53 has been measured, conducting GWASs on this biomarker across additional cohorts in the future would be feasible and could offer novel insights into the pathogenesis of AD. Using continuous endophenotypes like U-p53 in neurodegenerative diseases produces various benefits compared to traditional binary trait GWASs, such as increasing the statistical power of GWASs, reducing the disease heterogeneity which is often observed in complex diseases, and providing specific biological context for the associated genes [62].

### Supplementary Information

**Supplementary Material 1.**

## Data Availability

The DIADEM_V2_08_30_22.csv, ADNI_GO_2_OmniExpress.zip, ADNI_GO2_2nd.tar.gz, Microarray Gene Expression Profile Data.csv, and ADNIMERGE.csv files from the ADNI cohort are available on the https://ida.loni.usc.edu/ web portal via a personal account. The AD gene program file from the Karthik A. Jagadeesh et al. study [38] can be found on https://alkesgroup.broadinstitute.org/LDSCORE/Jagadeesh_Dey_sclinker/gene_scores/gene_scores/disease_progression_programs/Alzheimers/. The brain single-cell multimodal dataset is available on the GEO website via the accession code GSE214637. The brain single-cell RNA-seq dataset used for validation of the candidate genes is found on the GEO website via the accession code GSE174367. Other datasets used and/or analyzed during the current study are available from the corresponding author on reasonable request.
